# St. Louis College of Physicians and Surgeons

**Published:** 1888-07

**Authors:** 


### St. Louis College of Physicians and Surgeons—Dental Department.

The commencement exercises of the Dental Department of the St. Louis College of Physicians and Surgeons were held at Memorial Hall, St. Louis, Mo., March 3, 1888.

The degree of D.D.S. was conferred on the following graduates of the Dental Class by Professor Louis Bauer, Dean:

| NAME. | RESIDENCE. | NANE. | RESIDENCE. |
|---|---|---|---|
| George E. Beal | Pennsylvania. | Albert H. Hammen | Missouri. |
| John C. Cave | Nebraska. | William G. Lange | Canada. |
| Henry A. Collins | Illinois. | Carl Summa (*ad eundem*) | Missouri. |
| Peter H Eispoppel | Illinois. | | |

